# Giant pedunculated hepatic hemangioma accompanied by a 10‐year history of taking oral contraceptive: A case report and literature review

**DOI:** 10.1002/ccr3.8995

**Published:** 2024-05-26

**Authors:** Pirouz Samidoust, Maziar Moayerifar, Maede Mohammadian, Athar Zamani, Maryam Jafari, Mani Moayerifar, Fakhrieh Kalavari, Mahta Foroughifar

**Affiliations:** ^1^ Razi Clinical Research Development Unit, Razi Hospital Guilan University of Medical Sciences Rasht Iran; ^2^ Department of Vascular Surgery Razi Hospital, Guilan University of Medical Sciences Rasht Iran; ^3^ Department of Radiology, Shohada Hospital Shahid Beheshti University of Medical Sciences Tehran Iran; ^4^ Department of Pathology and Laboratory Medicine Guilan University of Medical Sciences Rasht Iran; ^5^ Student Research Committee, School of Medicine, Anzali International Campus Guilan University of Medical Sciences Rasht Iran; ^6^ Student Research Committee, School of Medicine Guilan University of Medical Sciences Rasht Iran

**Keywords:** case report, giant hemangioma, liver, oral contraceptive

## Abstract

**Key Clinical Message:**

Giant pedunculated hepatic hemangiomas, mostly seen in women, are considered a rare type of giant hepatic hemangioma, with challenging diagnosis. Unlike other types of liver hemangiomas, they can manifest different kinds of symptoms, and are prone to life‐threatening manifestations like rupture or torsion.

**Abstract:**

Hemangioma is the most common benign liver primary tumor. Hepatic hemangioma >4 cm (some studies suggest >10 cm) is referred to as a giant hemangioma. Although hepatic hemangioma does not manifest symptoms in most cases, a giant hepatic hemangioma can manifest different kinds of symptoms. Giant pedunculated hepatic hemangiomas are considered a rare type of giant hepatic hemangioma, with challenging diagnosis, as the thin pedicle could be hard to be detected on imaging. A 41‐year‐old woman was admitted to our hospital, with dull discomfort of the right upper quadrant and epigastric region and early satiety for the past 7 months, with the history of taking oral contraceptive (OCP) for 10 years. Ultrasound and computed tomography revealed a 130 × 124 × 76 mm solid mass, with central cystic lesion, located in the midline of the epigastric region, attaching to the inferior surface of the third segment of the left lobe of the liver. Due to the potential risk for torsion, and rupture of the hemangioma, the management of the patient proceeded to surgical excision. Pathological examination of the specimen confirmed the diagnosis of hepatic hemangioma. Giant pedunculated hepatic hemangioma is a rare benign tumor. It demonstrates higher incidence rate in women, as some hemangiomas have estrogen receptors, and estrogen can lead to endothelial cell proliferation and organization in vascular structure. Most hemangiomas do not express any symptoms; therefore, no treatment is needed except for the patients who manifest symptoms, or in giant pedunculated hemangiomas, as they are prone to rupture or torsion. In this review most cases were female, and most of them presented with abdominal pain, in most cases the tumor located in the left lobe of the liver. Almost all the reviewed cases underwent surgery. Giant hepatic hemangioma is a differential diagnosis of palpable mass, or other symptoms of the right upper quadrant, and epigastric region specially in women taking OCP. Imaging is needed to rule out these tumors, and most often, pedunculated hemangioma is harder to be defined on imaging. It requires surgery because of the risk of acute problems, such as torsion and rupture.

## Graphical abstract

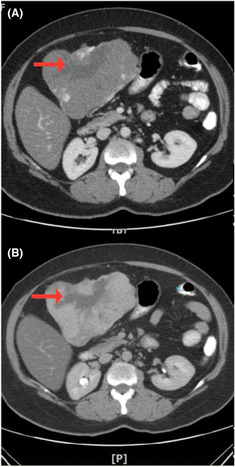

## 1 INTRODUCTION

Hemangioma is the most common benign liver primary tumor, which is believed to be a vascular malformation or hamartoma, with a frequency of 0.4%–20% in autopsy.^1^, ^2^ The condition could be detected across various age groups, particularly in middle‐aged women, and assumed to be related to hormone exposure. It is often located in the posterior segment of the liver right lobe. In most cases, hepatic hemangioma is asymptomatic and small, detected incidentally during abdominal imaging.^3^ Therefore, there is no need for treatment or radiological follow‐up except for giant hepatic hemangioma. Hepatic hemangioma >4 cm (some studies suggest >10 cm) is referred to as a giant hemangioma (GHH).^2^, ^4^, ^5^ A giant hepatic hemangioma can manifest different kinds of symptoms like pain and discomfort of the right upper abdomen, anorexia, palpable mass, obstructive jaundice, gastric outlet obstruction and bleeding, consumption coagulopathy, nausea, vomiting, and thrombocytopenia, which are characteristic of Kasabach‐Merritt syndrome.^3^ Giant pedunculated hepatic hemangiomas (GPHH) are considered a rare type of giant hepatic hemangioma, with challenging diagnosis, as the thin pedicle could be hard to be detected on imaging.^6^, ^7^ In this article, we report a 41‐year‐old woman with pedunculated giant hepatic hemangioma, who underwent the surgical excision of the tumor.

## 2 CASE PRESENTATION

### 2.1 Case History

We report a case of 41‐year‐old woman who presented with dull discomfort of the right upper quadrant and epigastric region and early satiety for the past 7 months. Additionally, the patient had a history of dyspepsia and bloating 2–3 h after meals during the past 10 years. She mentioned taking oral contraceptive (OCP) almost for a decade. The patient had a past surgical history of cesarean section 22 and 11 years prior to her presentation. She had no other past medical, social (smoking, drug or alcohol use), or familial history.

### 2.2 Investigations

Physical examination revealed obese abdomen, with deep palpable abdominal mass, without tenderness or guarding in the epigastric and right upper quadrant region. The blood tests including the complete blood count were almost normal, except for a mild increase in alanine aminotransferase. The patient had a normal chest x‐ray.

The abdominal ultrasonography demonstrated a normal liver size with increased echogenicity of the hepatic parenchyma, compatible with Grade 2 fatty liver disease. Ultrasound also revealed a 130 × 124 × 76 mm solid mass, with central hypoechoic lesion, located in the midline of the epigastric region, inclined to the right side. No pathologies were seen in the kidneys, pancreas and the spleen.

**FIGURE 1:**
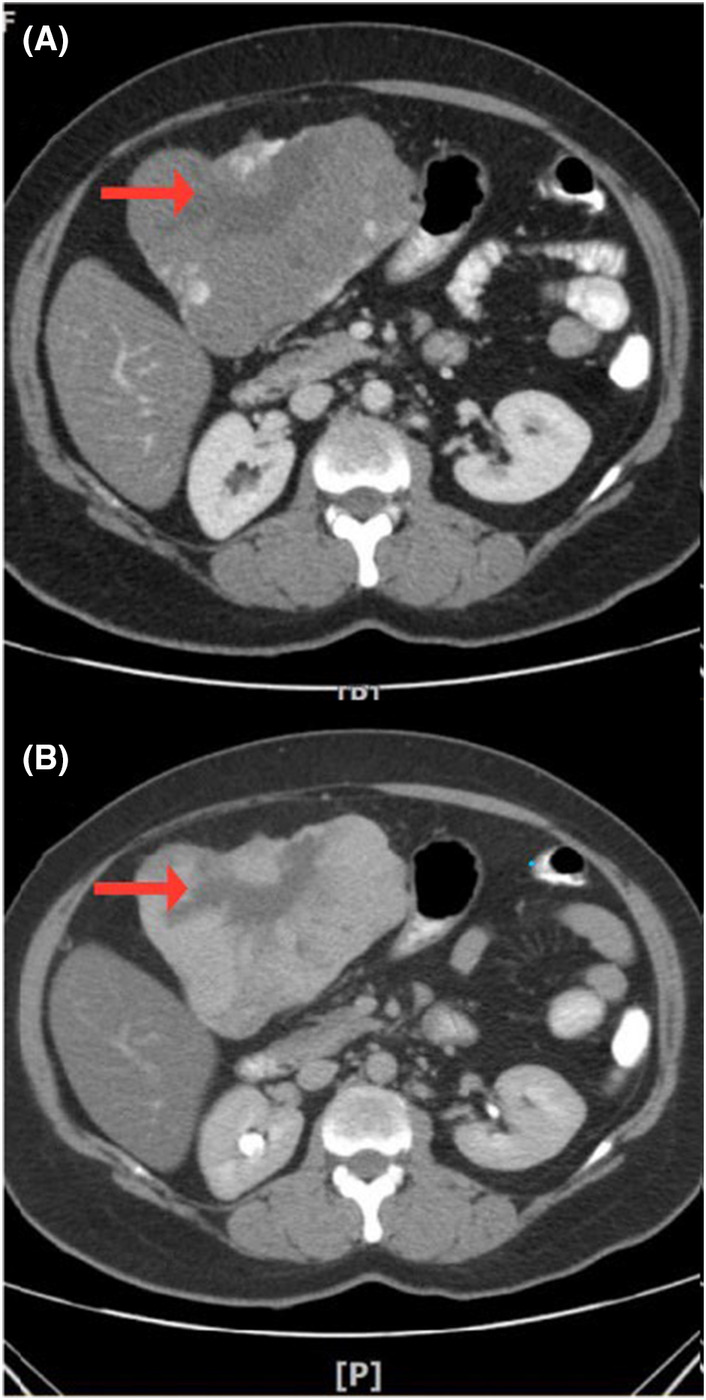
The red arrow indicates the probable central hemorrhage (A) portal phase of CT scan: Intense nodular marginal enhancement is noticed. (B) Latent phase of CT scan: a fill‐in pattern of enhancement is noticed, and most of it, except the inner areas, is hyper‐dense and have the same density as blood vessels, which recommends giant liver hemangioma.

**FIGURE 2:**
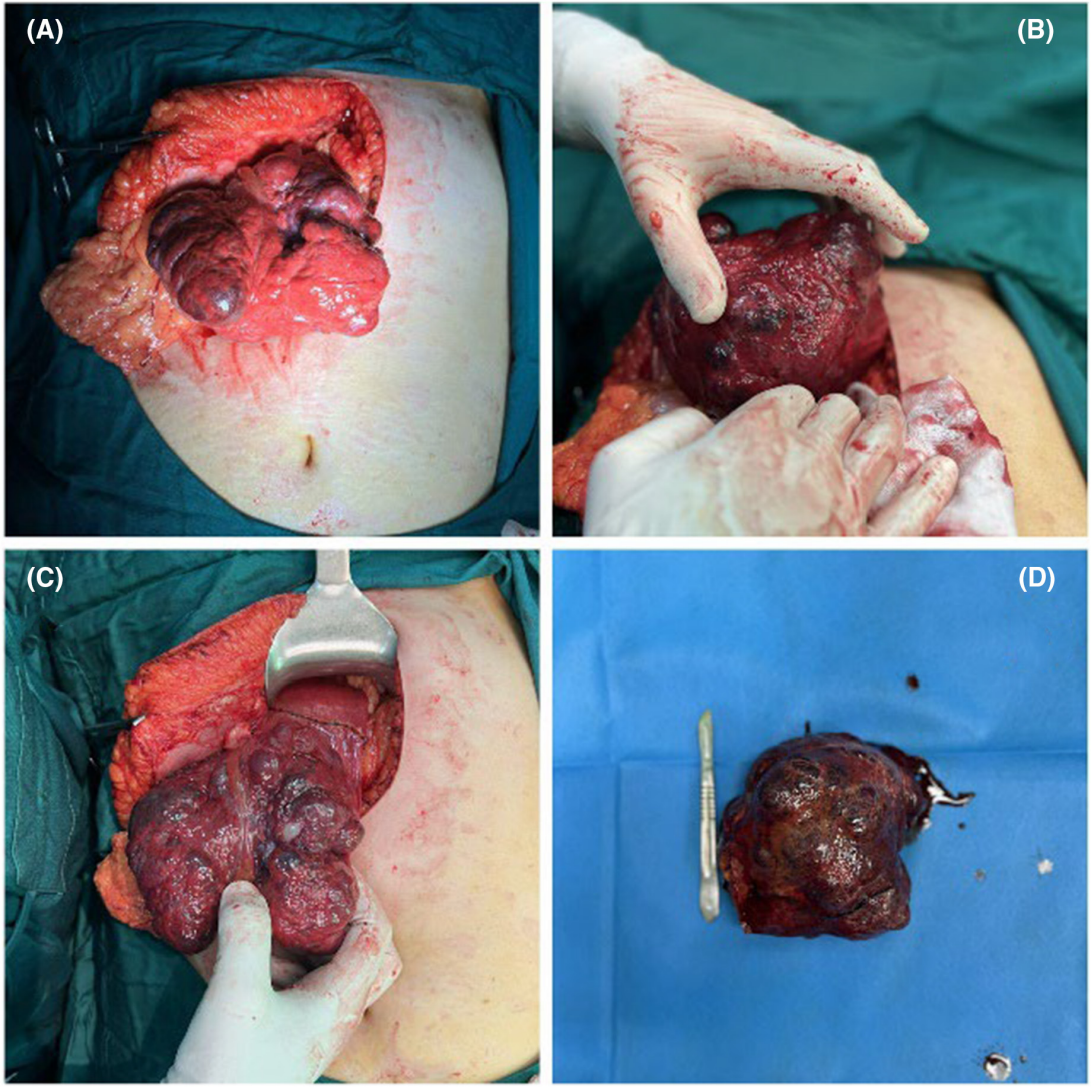
Intraoperative photograph of surgical field. **(**A, B, C) The giant pedunculated hemangioma originating from the left lobe of the liver. (D) The resected tumor.

The abdominopelvic contrast‐enhanced spiral computed tomography (CT) scan showed diffuse, lowered liver density, and sever fat infiltration. The pedunculated, huge, mass‐like lesion with 137 × 129 × 85 mm dimensions was attached to the inferior surface of the third segment of the left lobe of the liver. The mass prolapsed to the sub‐hepatic area, and peritoneal cavity, resulted in a pressure effect on the distal portion of the gastric body and shifting the transverse colon to the inferior. The mass consisted of a lobular margin and heterogeneous density with inner hypo‐dense zones, that were considered to be a probable central hemorrhage. In the portal phase of contrast injection findings, intense nodular marginal enhancement was noticed. In the latent phase, a fill‐in enhancement pattern was noticed, and most of it, except the inner areas, was hyper‐dense and had the same density as blood vessels, which recommended giant liver hemangioma. (Figure 1) Given the symptoms of the massive pedunculated hepatic angiomatosis, and the possibility of hemangioma rupture and torsion, surgery was the best option. (Figure 2). Opening the abdomen with Kocher incision a huge pedunculated mass was seen in left lobe of the liver. As the mass had a separated pedicle, proper hemostasis was obtained, and the mass was incised and detached from its pedicle. Intraoperative bleeding was almost 300 mL. After the operation the thromboprophylaxis was obtained by subcutaneous heparin twice daily for 7 days. The recovery period was uneventful and the patient was discharged home after 5 days.

## 3 RESULTS

**FIGURE 3:**
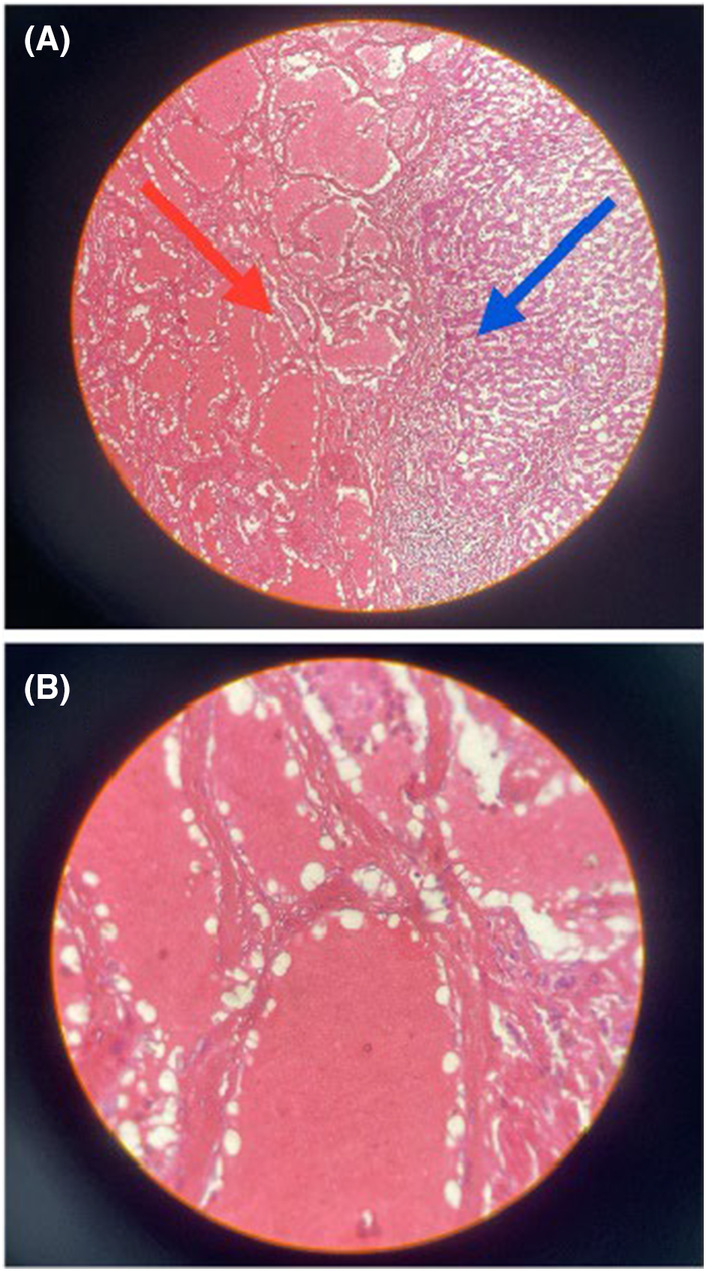
Pathological aspects: (A) Red arrow: proliferation of vascular channels, which are containing RBC'S. Blue arrow: steatotic hepatic parenchyma. H&E staining ×10 (B) the endothelial cells do not have atypia. No mitotic figure is appreciated. H&E staining ×40.

Pathologic examination of the specimen revealed a vascular neoplasm, which was surrounded by steatotic hepatic parenchyma. Neoplastic tissue composed of circumscribed proliferation of multiple thin‐walled and dilated vascular channels containing red blood cells. The endothelial cells of vascular channels did not have atypia. Detecting focal thrombi, and hyalinization, without any signs of mitotic activity were compatible with the diagnosis of hemangioma. (Figure 3).

On third month follow‐up, the patient did not have any complaints, and all of the patient's symptoms had been resolved.

## 4 DISCUSSION

Giant pedunculated hepatic hemangioma is a rare benign tumor, with only 27 previous case reports in the English literature. There is not a consensus on defining the size of giant hepatic hemangioma, as some researchers believe that a hepatic hemangioma larger than 5 cm is a giant hemangioma, and some others believe that a tumor larger than 10 cm is a giant one.^1^ Earlier studies of hepatic hemangioma demonstrated different prevalence between males and females, and revealed higher incidence rate in woman, since some hemangiomas have estrogen receptors. Containing estrogen receptors facilitate hemangioma's growth by taking estrogen‐containing drugs like OCP, or during pregnancy. Hemangiomas are vascular malformation, and estrogen can lead to endothelial cell proliferation and organization in vascular structure.^1^, ^3^, ^8^ Although most hepatic hemangiomas do not express any symptoms, some affected patients experience symptoms like abdominal pain, early satiety, and even life‐threatening symptoms, which are associated with certain conditions like Kasabach‐Merritt syndrome.^3^ As the most hemangiomas do not express any symptoms, no treatment is needed except for the patients who are symptomatic, or in giant pedunculated hemangiomas, because they are prone to rupture or torsion.^4^, ^9^ Surgery, particularly is indicated in the case of progressive abdominal symptoms, spontaneous rupture, rapidly enlarging lesions, Kasabach‐Merritt syndrome and suspicion of malignancy.^2^ Diagnosing giant pedunculated hepatic hemangioma can be challenging, as the slender pedicle may not be visible on images. Surgical treatment is consistently recommended for pedunculated hemangiomas due to their propensity for torsion and rupture.^9^

Table 1 reviews 15 cases of a pedunculated type of hepatic hemangioma. The mean age of cases was 46 years old. The frequency of pedunculated hepatic hemangioma in females and males was 53% and 47%, respectively. Most of the cases presented with abdominal pain or other non‐specific gastrointestinal symptoms including dyspepsia, nausea, vomiting, or bile duct obstruction, which can be related to the large size of the tumor and extrahepatic development. Three out of 15 cases were asymptomatic, and 3 cases manifestation was palpable mass, like ours. However, Hemangioma mainly originate from posterior segment of the right lobe of the liver, giant hepatic hemangioma in most cases arise from left lobe. In more than half of the cases, the size of the giant hepatic hemangioma was more than 10 cm. Due to the mobile or long pedicle of hemangioma, torsion can lead to acute complications; therefore, surgery is indicated in these patients, and almost all cases in this review underwent surgical removal of the mass. One article reports coincidence of taking OCP and the diagnosis of giant hepatic hemangioma, same as our case.

**TABLE 1:** Fifteen cases of giant pedunculated hepatic hemangiomas described in the literature.

	Sex/age	Clinical symptoms	Accompaniment	Liver lobe	Pathological size (cm)	Radiological exams	Liver enzymes	Treatment
1	Male/26^9^	Abdominal pain	Negative	Not Applicable	13	CT	Elevated	Surgery
2	Female/58^10^	Abdominal pain	Negative	Right	13.2	CT	Normal	Surgery
3	Female/63^11^	Abdominal distention and pain	Negative	Left	6.4 × 7	MRI, endoscopy	Not Applicable	Surgery
4	Male/48^7^	Abdominal pain, diarrhea, vomiting	Negative	Left	20 × 9 × 16	US, CT, MRI	Mild increase in gamma‐glutamyl transferase	Surgery
5	Female/45^12^	Abdominal pain, nausea, vomiting, and loss of appetite	History of OCP taking	Left	10.5 × 8.8 × 7	US, CT, abdominal x‐ray	Normal	Surgery
6	Female/51^6^	Asymptotic	Negative	Left	13.2	US, NCCT, MDCT, MRI	Normal	Surgery
7	Male/31^13^	Right iliac fossa pain	Appendicitis	Right	10 × 12 × 15	US, Abdominal x‐ray	Not Applicable	Surgery
8	Female/28^14^	Palpable mass	Negative	Not Applicable	18.0 × 9.4 × 5.2	US, CT, MRI	Not Applicable	Surgery
9	Male/35^15^	Palpable mass, abdominal discomfort rectorrhagia	Negative	Left	12 × 8 × 4	CT, esophago‐duodeno‐gastroscopy colonoscopy	Normal	Surgery
10	Female/63^16^	Abdominal pain	Hemochromatosis, previous hemangioma	Right	14 × 12.5	US, CT, MRI	Normal	Surgery
11	Female/69^17^	Asymptotic	Negative	Right	6	CT, US, MRI, angiography	Not Applicable	Surgery
12	Female/49^18^	Asymptotic	Negative	Left	10	CT, MRI, angiography	Not Applicable	Not Applicable
13	Male/42^19^	Palpable mass	Previous hemangioma	Left	5 × 4	US, CT, MRI	Not Applicable	Surgery
14	Male/36^19^	Asymptotic	Hepatic nodule	Left	5 × 4	US, CT, MRI, angiography	Normal	Surgery
15	Male/45^20^	Abdominal pain	Negative	Left	13	CT	Not Applicable	Surgery

## 5 CONCLUSION

In conclusion, giant hepatic hemangioma should be taken into consideration as a differential diagnosis in the case of pain, palpable mass, or other symptoms of the right upper quadrant, and epigastric region specially in women taking OCP, as OCP could result in hemangioma's growth. CT scan, MRI, and ultrasound imaging are needed to rule out these tumors. Most often, pedunculated hemangioma is hard to be defined on imaging, and has a higher risk of acute problems due to its thin pedicle. Therefore, it requires surgery because of the risk of torsion and rupture.

## AUTHOR CONTRIBUTIONS

**Pirouz Samidoust:** Supervision; validation; visualization; writing – original draft; writing – review and editing. **Maziar Moayerifar:** Validation; visualization; writing – original draft; writing – review and editing. **Maede Mohammadian:** Writing – original draft; writing – review and editing. **Athar Zamani:** Validation; visualization; writing – original draft; writing – review and editing. **Maryam Jafari:** Writing – original draft; writing – review and editing. **Mani Moayerifar:** Validation; visualization; writing – original draft; writing – review and editing. **Fakhrieh Kalavari:** Validation; visualization; writing – original draft; writing – review and editing. **Mahta Foroughifar:** Writing – original draft; writing – review and editing.

## FUNDING INFORMATION

There was no funding, financial support, and sponsorship for this article.

## CONSENT

Written informed consent was obtained from the patient to publish this report in accordance with the journal's patient consent policy.

## Data Availability

The data that supports the findings of the study are available on request from the corresponding author.
